# A Twist in Perception: A Case of an Eight-Year-Old Female With Alice in Wonderland Syndrome

**DOI:** 10.7759/cureus.60182

**Published:** 2024-05-13

**Authors:** Suchita Manwar, Bhagyesh Sapkale, Truptesh Patil, Anjali Vagga

**Affiliations:** 1 Medicine, Jawaharlal Nehru Medical College, Datta Meghe Institute of Higher Education and Research, Wardha, IND; 2 Biochemistry, Jawaharlal Nehru Medical College, Datta Meghe Institute of Higher Education and Research, Wardha, IND

**Keywords:** diagnostic challenges, multisensory perception, migraine prophylaxis, tachysensia, auditory abnormalities, visual anomalies, perception distortions, pediatric case, aiws, alice in wonderland syndrome

## Abstract

The article aims to explore the challenges involved in diagnosing and managing Alice in Wonderland Syndrome (AIWS) in pediatric cases, focusing on an eight-year-old female with perceptual distortions affecting vision, hearing, and time perception. AIWS, a rare neurological phenomenon, manifests as distortions in the perception of the body and external stimuli. The lack of established diagnostic criteria, particularly in the pediatric population, complicates accurate identification. The presented case illustrates visual anomalies, auditory abnormalities, and tachysensia, emphasizing the multisensory nature of AIWS. The temporal association with underlying causes, such as migraines and viral infections, highlights the need for a comprehensive evaluation. The Acharya Vinoba Bhave Rural Hospital management approach involves a systematic assessment, identification of underlying chronic conditions, and targeted treatment. Migraine prophylaxis, utilizing prescription drugs and a low-tyramine diet, plays a central role. The limited use of antipsychotics underscores the neurological origin of AIWS. The article contributes valuable insights into pediatric AIWS, advocating for further research and awareness. The article also aims to highlight the lack of established diagnostic criteria for AIWS, particularly in the pediatric population, and to present a systematic management approach based on a specific case study. The multidisciplinary collaboration, regular follow-ups, and patient education constitute a comprehensive approach to enhance understanding and alleviate symptoms in AIWS cases.

## Introduction

Alice in Wonderland syndrome (AIWS) is a rare neurological phenomenon characterized by distortions in perception, impacting various sensory modalities such as vision, touch, and hearing [1]. The symptoms of changed body image are linked to Alice in Wonderland syndrome (AIWS). A shift in visual perception is thought to cause an inability to distinguish between the correct size of objects outside the body or body components [1,2]. Body schema, a confused sense of time, and distorted eyesight are characteristics of AIWS [3]. Patients with AIWS experience distorted vision, which causes objects to appear smaller (micropsia), larger (macropsia), farther away (teleopsia), or closer (pelopsia) than they are [4]. A few additional senses, in addition to vision, may also be affected by AIWS [5]. Our immediate impression of time is significantly altered by AIWS, which is perhaps its most intriguing and little-known characteristic [5]. Head trauma, headaches, and viral encephalitis from Epstein-Barr virus infection have all been associated with AIWS [1,6], while the specific cause of the illness is still unknown.

Furthermore, it is postulated that abnormal electrical activity in the brain areas in charge of processing texture and vision in AIWS may lead to irregular blood circulation [7]. Although it can affect adults and teenagers as well, children are the ones who experience AIWS the most commonly [8]. The temporal association between the underlying cause of AIWS development and the onset of perceptual distortions aligns with previous literature linking AIWS to conditions such as migraines, epilepsy, and viral infections [1,8]. The symptoms of true AIWS are limited to those linked to alterations in an individual's perception of their body. Conversely, we designate as an "AIWS-like condition" all symptoms of altered perceptions of time, vision, hearing, touch, or other external senses [9].

## Case presentation

We present the case of an eight-year-old female child who was brought to Acharya Vinoba Bhave Rural Hospital (AVBRH) with a peculiar set of symptoms indicative of Alice in Wonderland Syndrome (AIWS). The child's parents sought medical attention late in the afternoon after noticing pronounced visual anomalies, auditory abnormalities, and a loss of the sense of time experienced by the child. The daytime onset of symptoms and their progression throughout the day prompted concern, leading to the decision to seek medical evaluation. The onset of the underlying cause of AIWS, potentially linked to migraines, epilepsy, or a viral infection, began on January 21, 2024. In the days or weeks leading up to the presentation, the child experienced occasional subtle sensory distortions, escalating to more intense and frequent perceptual disturbances on the day of admission. AIWS is a rare neurological phenomenon characterized by distortions in perception, affecting various sensory modalities such as vision, touch, and hearing. The episodic nature of AIWS and its diverse symptoms pose diagnostic challenges, especially in pediatric cases.

Clinical findings

**Figure 1:**
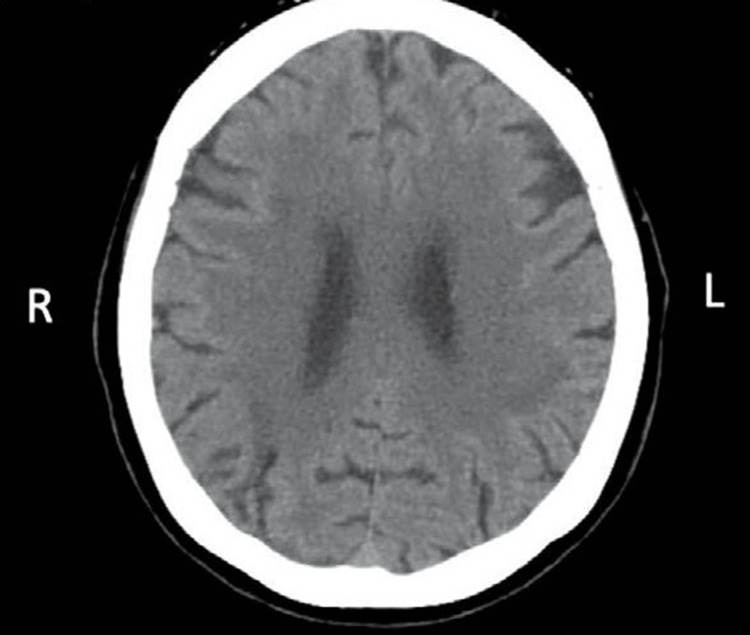
Computed tomography image of the brain Computed tomography revealed a low intensity in the surrounding cortex of the brain.

Upon admission to AVBRH, a thorough diagnostic evaluation was initiated to elucidate the underlying cause of the eight-year-old female child's symptoms suggestive of Alice in Wonderland Syndrome (AIWS). A comprehensive neurological examination was conducted to assess sensory perception, motor function, and coordination, with a particular focus on identifying any signs indicative of neurological abnormalities. Subsequently, computed tomography (CT) imaging of the brain was performed, revealing a low intensity in the surrounding cortex, prompting further investigation into potential neurological irregularities associated with AIWS as shown in Figure 1. Additionally, electroencephalography (EEG) could have been conducted to assess brain electrical activity and detect abnormalities indicative of epilepsy. Laboratory investigations, including blood tests and possibly a lumbar puncture, were undertaken to evaluate for signs of infection, metabolic abnormalities, or systemic inflammation. The patient exhibited visual anomalies consistent with AIWS, including macrosomatognosia and microsomatognosia. She reported experiencing metamorphopsias, commonly known as Lilliputian hallucinations, where objects appeared smaller (micropsia) or larger (macropsia) than their actual size. Additionally, the child described vivid zoopsias, hallucinating swarms of small or solitary larger animals, a phenomenon associated with AIWS. The child presented with auditory abnormalities, including misreading everyday noises and heightened sensitivity to delicate sounds. Pitch and tone distortions, as well as hearing indistinct and unusual noises, were noted.

Furthermore, there was evidence of tachysensia, a loss of the sense of time, commonly observed in AIWS. The timeline of events in the case is shown in Table 1.

**Table 1:** Timeline of events in the case AIWS: Alice in Wonderland Syndrome

Time	Event
21 Jan 2024	The underlying cause of AIWS develops (e.g., migraine, epilepsy, viral infection).
Days/weeks prior to the presentation	The child may experience occasional, subtle sensory distortions.
Day of presentation	The child experiences more intense and frequent perceptual distortions.
Morning	The child experiences visual anomalies, Macrosomatognosia, Microsomatognosia, Metamorphopsias (Lilliputian hallucinations), and Zoopsias.
Mid-morning	The child experiences auditory abnormalities, misreading of common noises, heightened sensitivity to delicate sounds, pitch and tone distortions, and hearing indistinct and unusual noises.
Afternoon	The child experiences tachysensia (loss of sense of time).
Late afternoon	Parents seek medical attention.
Evening	The child arrives at Acharya Vinoba Bhave Rural Hospital for evaluation.
Following days	Diagnostic workup to identify the underlying cause of AIWS.
Treatment begins	Appropriate treatment for AIWS and underlying conditions was initiated.
Recovery	The child's symptoms gradually improve and resolve with treatment.

The lack of established diagnostic criteria for AIWS, particularly in the pediatric population, led to difficulty identifying the condition. The child's reluctance and challenges in articulating her symptoms further complicated the diagnostic process. The diagnosis of AIWS was considered after ruling out other possibilities and considering the daytime onset of symptoms, along with additional migraine-associated features. Differential diagnosis involved ruling out psychosis and distinguishing AIWS from other conditions causing hallucinations. Most notably, the child displayed an awareness that her hallucinations were not genuine, aiding in the distinction.

Management

In managing the case of Alice in Wonderland Syndrome (AIWS) in our pediatric patient, a systematic and thorough approach was adopted at Acharya Vinoba Bhave Rural Hospital. The initial step involved a comprehensive evaluation upon admission, encompassing a detailed medical history, physical examination, and neurological assessments. This process aimed to unravel the intricacies of perceptual distortions associated with AIWS in the eight-year-old female child. Identifying underlying chronic conditions became a pivotal focus, given the episodic nature of AIWS. Special attention was paid to correlating the patient's symptoms with potential triggers, particularly emphasizing features associated with migraines.

Migraine prophylaxis played a central role in the management approach, utilizing prescription drugs known for their efficacy in preventing migraines. Anticonvulsants, beta-blockers, calcium channel blockers, and antidepressants were considered based on the child's unique clinical presentation. Treatment was strategically directed towards the suspected underlying conditions, especially when AIWS symptoms were linked to other chronic diseases. Prescription medications, including antivirals, antibiotics, and antiepileptics, were tailored to the identified etiology, with observed symptom relief upon successful treatment of the underlying disease.

Antipsychotics, recognized for their limited effectiveness in addressing AIWS symptoms, were sparingly utilized. The primary focus remained on medications targeting suspected underlying conditions and the implementation of a low-tyramine diet as part of the migraine prophylaxis strategy. The complexity of AIWS necessitated a multidisciplinary approach involving collaboration between neurologists, pediatricians, and other specialists. Regular follow-ups facilitated adjustments to the treatment regimen based on the child's response to interventions.

Emphasis was placed on patient education and support throughout the management process. The child and her caregivers were informed about the nature of AIWS, its episodic characteristics, and the crucial role of adherence to the prescribed treatment plan. Continuous support was provided to address any concerns or challenges that emerged during the course of treatment. In conclusion, the management of AIWS in this pediatric case embraced a comprehensive, individualized, and collaborative approach. By targeting underlying chronic conditions and implementing migraine prophylaxis, the medical team aimed to alleviate symptoms, enhance the child's quality of life, and contribute valuable insights to the ongoing understanding of this unique neurological phenomenon. The management approach is described in Table 2.

**Table 2:** The management approach for AIWS: Alice in Wonderland Syndrome

Management Approach	Description
Comprehensive evaluation	Medical history, physical examination, neurological assessments.
Identification of underlying chronic conditions	Migraines and other chronic diseases.
Migraine prophylaxis	Prescription drugs (anticonvulsants, beta-blockers, calcium channel blockers, antidepressants), low-tyramine diet.
Antipsychotics	Limited use, only if other treatments fail.
Multidisciplinary approach	Neurologists, pediatricians, and other specialists.
Regular follow-ups	Adjustments to treatment based on response.
Patient education and support	Information about AIWS, adherence to the treatment plan, and address concerns.

## Discussion

Alice in Wonderland syndrome (AIWS) is a rare neurological phenomenon characterized by distortions in perception, impacting various sensory modalities such as vision, touch, and hearing [1]. The presented case of an eight-year-old female with AIWS underscores the challenges in diagnosing and managing this condition, particularly in the pediatric population. The lack of established diagnostic criteria, coupled with the episodic and diverse nature of AIWS symptoms, contributed to the complexity of the diagnostic process. The patient exhibited classic visual anomalies associated with AIWS, including macrosomatognosia, microsomatognosia, metamorphopsias (Lilliputian hallucinations), and zoopsias. These symptoms were accompanied by auditory abnormalities and tachysensia, further highlighting the multisensory nature of AIWS in this case. The episodic progression of symptoms, with occasional subtle distortions preceding more intense perceptual disturbances, posed a diagnostic challenge, especially given the child's difficulty articulating her experiences.

The temporal association between the underlying cause of AIWS development and the onset of perceptual distortions aligns with previous literature linking AIWS to conditions such as migraines, epilepsy, and viral infections [1,8]. The case highlights the importance of considering AIWS in diagnosing pediatric patients with unusual sensory experiences, emphasizing the need for a comprehensive evaluation. The management approach adopted at Acharya Vinoba Bhave Rural Hospital focused on a systematic assessment, identification of underlying chronic conditions, and targeted treatment. Migraine prophylaxis played a central role, reflecting the association between AIWS and migraines in the literature [2,7]. The use of prescription drugs, including anticonvulsants, beta-blockers, calcium channel blockers, and antidepressants, demonstrated a tailored approach based on the child's unique clinical presentation. The limited use of antipsychotics underscored the understanding that AIWS primarily stems from neurological rather than psychiatric origins.

The multidisciplinary collaboration involving neurologists, pediatricians, and other specialists ensured a comprehensive and individualized management strategy. Regular follow-ups allowed for adjustments to the treatment regimen based on the child's response. Patient education was crucial in promoting adherence to the prescribed treatment plan and addressing concerns. In comparing this case to previous reports, several commonalities emerge. A case presented by Weissenstein et al. described a six-year-old child with AIWS, emphasizing the association with migraines and the importance of careful evaluation [8]. Another case by George and Bernard highlighted complex hallucinations in a 13-year-old with migraines, further supporting the link between AIWS and migraine pathology [9]. The present case adds to the body of evidence supporting the role of migraine prophylaxis in managing AIWS symptoms.

## Conclusions

In conclusion, this case report details the challenges in diagnosing and managing Alice in Wonderland Syndrome (AIWS) in an eight-year-old female. AIWS, a rare neurological phenomenon affecting perception, poses diagnostic complexities, especially in pediatric cases. The child exhibited visual and auditory anomalies and a distorted sense of time. The temporal association with the underlying cause's development highlighted the importance of considering AIWS in pediatric patients with unusual sensory experiences. A systematic, multidisciplinary approach was adopted, including comprehensive evaluation and migraine prevention. The limited use of antipsychotics underscored the neurological origin of AIWS. The article aims to elaborate on the challenges involved in diagnosing and managing AIWS in pediatric cases, highlighting the lack of established diagnostic criteria. Suggestions for future research include the development of standardized diagnostic criteria for AIWS, particularly tailored to pediatric patients, to facilitate accurate and timely diagnosis. Longitudinal studies could investigate the natural history of AIWS in pediatric populations, including the frequency and severity of symptoms, as well as the impact on quality of life and psychological well-being. Further research could explore the underlying pathophysiological mechanisms of AIWS and potential therapeutic interventions, including the efficacy of migraine prophylaxis and other treatment modalities. Collaborative efforts between neurologists, paediatricians, psychologists, and other specialists may also be beneficial in advancing understanding and management strategies for AIWS in pediatric patients.
